# Unlocking γδ T cell power: pathways that boost cancer defense

**DOI:** 10.1186/s43556-023-00168-6

**Published:** 2024-02-05

**Authors:** Yuhao Yao, Zhi Zong, Long Zhang

**Affiliations:** 1 International Biomed-X Research Center, Second Affiliated Hospital of Zhejiang University School of Medicine, Zhejiang University, Hangzhou, China; 2 MOE Key Laboratory of Biosystems Homeostasis & Protection and Innovation Center for Cell Signaling Network, Life Sciences Institute, Zhejiang University, Hangzhou, Zhejiang 310058 China; 3 Cancer Center, Zhejiang University, Hangzhou, China

Recently, Mamedov et al. published their new study in Nature [1], where they used genome-wide CRISPR screening of target cancer cells to identify pathways that regulate γδ T cell killing and BTN3A cell surface expression. γδ T cells, also known as gamma-delta T cells, are a subset of T cells in the immune system. The study’s findings demonstrate that the levels of BTN3A on the cell surface and the activation of γδ T cells can be controlled through various mechanisms, including transcriptional regulation, post-translational modifications, and the process of membrane trafficking.

γδ T cells stand out as promising candidates for advancing tumor immunotherapy. Their unique capacity to activate both innate and adaptive immunity sets them apart. In contrast to the specific molecular responses elicited by the classic αβ T cell receptor, γδ T cells stimulate innate immunity more broadly. This characteristic makes γδ T cells exceptionally versatile for anti-tumor applications, facilitating the use of both autologous and allogeneic cell therapies [2]. Vγ9Vδ2 T cells exhibit a consistent specificity for the BTN2A1-BTN3A1-BTN3A2 complex, and elevated expression of BTN3A1 (CD277) can be observed within malignant tumors [3]. The mevalonate pathway mediates the phosphorylation of antigen metabolites and recruits BTN3A1, allowing BTN2A1 to directly bind to Vγ9 [4], activate the triplex complex, and activate Vγ9Vδ2 T cells [5]. Despite these promising insights, the dynamic relationship between Vγ9Vδ2 T cells and cancer cells remains incompletely understood. Emphasizing the unique capacity of γδ T cells to activate both innate and adaptive immunity is crucial. Additionally, contrasting these cells with αβ T cells highlights the broader cancer-killing activity of γδ T cells, which is not confined to patient-specific neoantigens or the human leukocyte antigen background. This broader scope enhances the applicability of γδ T cells in anti-tumor strategies, underscoring the need for a deeper understanding of the factors governing their interactions with cancer cells.

To uncover genes responsible for the destruction of cancer cells by human Vγ9Vδ2 T cells, Mamedov et al. used CRISPR to create a genome-wide pool of knockout (KO) Daudi-Cas9 cells. Following treatment with zoledronic acid, these modified Daudi-Cas9 cells were cultured alongside primary Vγ9Vδ2 T cells that had been expanded from healthy donors. Finally, the sgRNA of surviving Daudi cells after co-culture was detected. The research successfully identified important regulators of interactions between cancer cells and Vγ9Vδ2 T cells, which improve survival rates. These regulators included specific genes involved in the butyrophilin complex, enzymes involved in the mevalonate pathway, transporter genes, gene activators, and surface proteins. This discovery has substantial potential for the advancement of cancer treatment strategies. Gene set enrichment analysis (GSEA) identified multiple metabolic pathways that, when knocked out, resulted in reduced survival during co-culture. Notably, the oxidative phosphorylation, tricarboxylic acid (TCA) cycle, and purine metabolism pathways from the Kyoto Encyclopedia of Genes and Genomes (KEGG) showed significant enrichment in this regard.

To assess the applicability of their findings beyond the Daudi cell line co-culture model and verify their consistency with real-world clinical data, Mamedov and colleagues created a gene signature using the co-culture screening outcomes. They then calculated gene signature scores for tumors across 33 different cancer types from The Cancer Genome Atlas, and analyzed the correlation between these scores and patient survival. Among the various tumor types analyzed, the most pronounced correlation was found in low-grade glioma tumors, where the gene signature associated with the TCA cycle from the KEGG pathway exhibited a particularly strong negative correlation. Furthermore, this study specifically ruled out the involvement of the TCR in the observed effects.

In order to gain a comprehensive understanding of the gene pathways that influence the levels of BTN3A on the cell surface, they performed a CRISPR screen to uncover the regulators of BTN3A surface expression. Among the notable findings with a false discovery rate (FDR) of less than 0.01 in the BTN3A expression assessment, 74% were in agreement with the results of the co-culture screening, where the FDR threshold was set at less than 0.05. This suggests a strong connection between factors influencing BTN3A surface expression and the effectiveness of Vγ9Vδ2 T cell-mediated target cell destruction. The toxic effect of Vγ9Vδ2 T cells on tumor cells depends largely on the level of BTN3A on the cell surface. The higher the BTN3A level, the stronger the cytotoxic effect (Fig. 1).

**Fig. 1:**
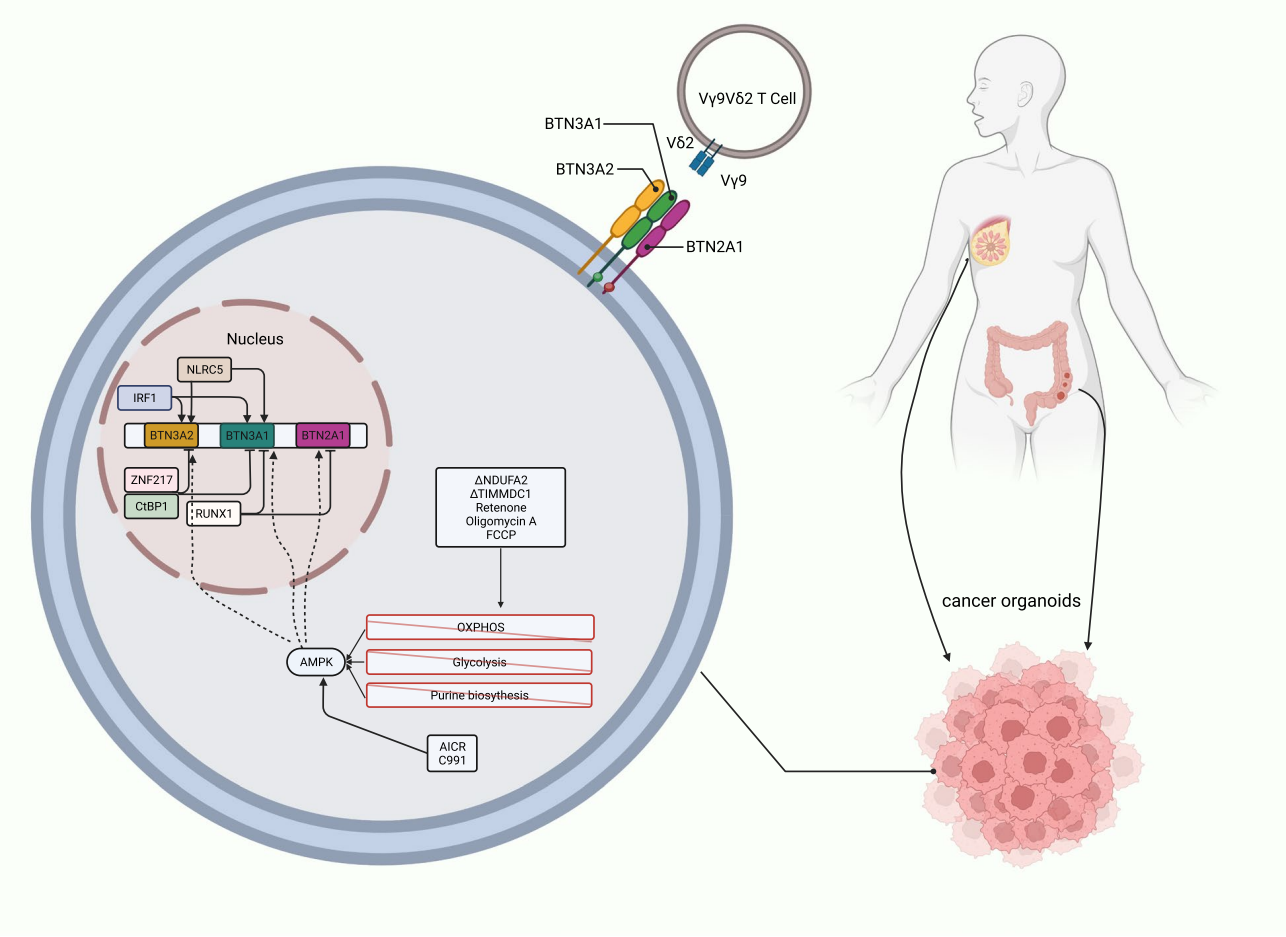
Model of regulation of the butyrophilin complex. Activation of the BTN2A1-3A1-3A2 complex through phosphoantigen metabolite engagement with BTN3A1 intracellular binding pocket. Genetic perturbations and inhibitors affecting metabolic pathways, specifically those involved in ATP production, were discovered to modulate BTN3A levels. This induction of BTN3A and BTN2A1 occurs during metabolic crises and relies on AMPK, ultimately enhancing Vγ9Vδ2 T cell receptor-mediated killing. endoplasmic reticulum (ER), endoplasmic reticulum; oxidative phosphorylation (OXPHOS), oxidative phosphorylation

While the influence of the mevalonate pathway on Vγ9Vδ2 TCR–BTN2A1/3A1 interactions via phosphoantigen levels is known, its role in controlling BTN3A surface abundance was previously unacknowledged. The expression of BTN3A is affected by multiple metabolic pathways. Previous studies have confirmed that the mevalonate pathway regulates this result; however, this was not thought to be achieved by regulating BTN3A levels. This study found that when the mevalonate pathway enzyme FDPS (farnesyl diphosphate synthase) was knocked out, the level of BTN3A on the cell surface increased. Additionally, knocking out related enzymes, such as the de novo purine synthesis pathway and Fe-S cluster synthesis, also increases BTN3A levels and enhances its ability to kill tumor cells. The de novo purine synthesis pathway involves adenosine monophosphate (AMP), which regulates AMPK and ATP synthesis (Fig. 1).

Furthermore, CUT&RUN analysis was used to determine whether IRF1 positively regulates the BTN3A1, BTN3A2, and BTN3A3 promoters associated with H3K4me3, and whether ZNF217 negatively regulates these promoters. These findings suggest a mutual regulation by IRF1 and ZNF217. The relationship between oxidative phosphorylation and BTN3A expression is complex and may be influenced by external culture conditions. Knocking out the electron transport chain complex I aligned with the co-culture screening results but contradicted BTN3A screening. Previous research has highlighted that altered ATP synthesis and NADH:NAD^+^ ratio affect BTN3A expression. Additional experiments have shown that BTN3A expression is energy-dependent; increased glucose and inhibition of glycolysis or specific complexes elevate surface BTN3A levels.

Certain small-molecule drugs that activate AMPK upregulate BTN3A expression. Metformin, an indirect AMPK agonist, modestly elevates BTN3A surface levels. This AMPK-driven increase was confirmed using two direct agonists, C991 and A-769662, in WT Daudi-Cas9 cells. Notably, colon and breast cancer organoids treated with AMPK agonists also exhibited increased surface BTN3A levels. However, knocking out AMPK without subjecting the cells to stress conditions did not induce BTN3A changes.

The research exploring the interaction between γδ T cells, particularly Vγ9Vδ2 T cells, and cancer cells represents a significant advancement in tumor immunotherapy. Notably, γδ T cells exhibit a unique dual activation of both innate and adaptive immunity, emphasizing their versatility for diverse anti-tumor applications. The study sheds light on the broad cancer-killing activity of γδ T cells, distinguishing them from αβ T cells and highlighting their potential beyond patient-specific neoantigens. Insights into the regulatory roles of BTN3A and the mevalonate pathway provide potential therapeutic targets, and the identification of key regulators through CRISPR screening offers avenues for precise interventions. Clinical correlations across various cancer types underscore the research’s translational relevance, while the discovery of AMPK’s role suggests promising directions for future drug development. Overall, this research significantly contributes to our understanding of γδ T cell interactions, opening new avenues for the development of targeted and effective immunotherapies with implications for various cancers.

## Data Availability

Not applicable.
